# Care-seeking behaviors and risk factors in women with postpartum urinary incontinence

**DOI:** 10.3389/fmed.2025.1710600

**Published:** 2025-12-01

**Authors:** Thamir Al-khlaiwi, Syed Shahid Habib, Sarah Muadi, Shuruq Alotaibi, Albandary Bin Hadba, Munirah Alshaqrawi, Deema Almuhaimel, Sarah Alshammari, Muhammad Iqbal

**Affiliations:** Department of Physiology, College of Medicine, King Saud University, Riyadh, Saudi Arabia

**Keywords:** postpartum urinary incontinence, prevalence, education, knowledge, employment

## Abstract

**Background:**

This study aimed to evaluate the attitudes and care-seeking practices of Saudi women, as well as to identify the risk factors associated with postpartum urinary incontinence (PPUI).

**Methods:**

A cross-sectional study was conducted among 813 postpartum Saudi Arabian women, selected using a convenience sampling method. Data were collected via an online questionnaire comprising sections on demographic information, the International Consultation on Incontinence Questionnaire–Urinary Incontinence Short Form (ICIQ-UI SF), the Incontinence Impact Questionnaire–Short Form (IIQ-7), and items assessing participants’ attitudes and practices related to seeking care.

**Results:**

The prevalence of PPUI among Saudi women was 29.6% (*n =* 241), with 15.8% (*n =* 38) reporting symptoms occurring several times a day. Over 20% of participants perceived that PPUI significantly interfered with their daily lives. Furthermore, 56.5% (*n =* 459) believed that urinary incontinence (UI) is a normal consequence of childbirth, and approximately two-thirds (67.3%) considered it a temporary condition that resolves over time. Among those experiencing PPUI, 66.4% did not seek medical care, while 34.9% reported seeking advice from their parents. Symptom severity was significantly associated with care-seeking, as 56.1% of those who sought care reported severe or extremely severe UI (*p* = 0.003; odds ratio = 4.595).

**Conclusion:**

This study identified a high prevalence of Saudi women who did not seek care for PPUI, with a positive correlation between seeking care and symptom severity. Additionally, a lack of accurate knowledge about PPUI and its risk factors was observed even among highly educated women, indicating a gap in health education and awareness.

## 1 Introduction

Urinary incontinence (UI) is a condition that affects women of all ages, cultures, and ethnicities worldwide. The global prevalence of UI varies widely, ranging from 4.8 to 58.4% across different populations (1). In Saudi Arabia, the prevalence ranges between 29 and 42.6%, and the majority of affected women do not seek medical assistance (2–4). In Oman, the prevalence of UI is 34.5%, but only 11.1% of women with symptoms have sought medical care (5). Postpartum urinary incontinence (PPUI) is a specific subtype of UI that occurs after childbirth, affecting approximately four out of 10 women (6). PPUI is defined as “Complaint of involuntary loss of urine experienced during the postpartum period and up to 12 months after delivery” as defined by the International Continence Society (7). PPUI is a broad term describing incontinence after delivery, and stress urinary incontinence can occur after delivery. Several types of UI have been identified, with stress urinary incontinence (SUI) being the most prevalent (8). SUI is typically associated with increased intra-abdominal pressure, which elevates bladder pressure and compromises its ability to retain urine (9). Since bladder control is primarily maintained by the pelvic floor muscles, any hormonal or anatomical changes during pregnancy and delivery can impair the function of these muscles, leading to UI (10). A prevalence of 35.9% has been reported in Türkiye, while a systematic review estimated a global PPUI prevalence of 33%, with rates of 31% following vaginal delivery and 15% following cesarean section (11, 12). Despite the clinical significance of PPUI, research on its prevalence, risk factors, and care-seeking behavior in Saudi Arabia remains limited.

Several risk factors trigger the development of PPUI, including being above the age of 40 with multiple births, use of oral contraceptives, cigarette smoking, type 2 diabetes mellitus, obesity, gestational age, number of previous vaginal deliveries, high body mass index (BMI), and a history of hysterectomy (13, 14). In Saudi Arabia, risk factors for UI among women include older age, urinary tract infections, parity, multiple vaginal deliveries, hypertension, asthma, and chronic cough (15). Additional factors specifically associated with PPUI include age, educational level, number of spontaneous vaginal deliveries, nocturia, and constipation. Pregnancy- and delivery-related variables are also known to significantly influence the risk of developing UI (11). Knowledge and awareness of PPUI among women are crucial factors for effective prevention and management. Poor communication, limited health education, and the influence of familial or cultural beliefs often contribute to the normalization of PPUI and act as barriers to seeking care (16). Additionally, healthcare professionals might not provide enough information on the issue, further contributing to a lack of awareness. In the Middle East, 80% of women with UI have never sought medical advice; a misunderstanding of the causes of UI, lack of awareness about available treatment options, embarrassment, and the assumption that UI is a normal part of aging were the most common barriers to seeking help. The belief that urinary incontinence may resolve spontaneously, the perception that it is a normal consequence of multiple births, and the concern that treatment might be costly, all contribute to delays in seeking medical care (17). Therefore, the present study aimed to evaluate the attitudes and care-seeking practices of Saudi women, as well as to identify risk factors associated with PPUI. The findings of our study will help nurses and healthcare professionals provide high-quality healthcare to postpartum women.

## 2 Methods

This observational, cross-sectional study was conducted between January 2023 and December 2023 using an online questionnaire to assess PPUI among Saudi women who have experienced PPUI symptoms within 1 year (7). A convenience sampling technique was employed to recruit adult Saudi women who met the inclusion criteria. Eligible participants included Saudi women who were currently or previously married and had experienced at least one childbirth. This included widowed and divorced women with a history of pregnancy and delivery. The exclusion criteria comprised non-Saudi women and married women without a history of childbirth. In addition, any woman who experienced urinary incontinence before pregnancy was excluded. The sample size was calculated using the formula *n =* Z^2^ × P(1 − P) / d^2^, where Z = 1.96 (95% confidence level), d = 0.05, and *p* = 30% postpartum UI prevalence, [(1.96)^2^ × 0.30 × (1–0.30) / (0.05)^2^ = 322]. To account for a 20% expected non-response rate, the final target sample size was 386. However, a total of 813 women responded and were included in the final analysis.

This study was approved by the Institutional Review Board of the College of Medicine Research Centre at King Saud University, Riyadh, Saudi Arabia (Ref: E-22-7062). Participants were informed that by voluntarily completing the questionnaire, they were providing their informed consent to participate in the study. Confidentiality and anonymity were ensured and maintained. All authors confirm that the study was conducted in accordance with the relevant guidelines and regulations outlined in the Declaration of Helsinki.

### 2.1 Data collection

Data were collected using three predesigned questionnaires. The first part was developed by the research team and included items on personal information, knowledge of PPUI and its risk factors, as well as attitudes and practices related to seeking care for PPUI. The remaining parts consisted of validated instruments, including a validated Arabic version of the International Consultation on Incontinence Questionnaire (ICIQ-UI-SF) (18, 19). This tool assesses the frequency of UI (never or once a week, two to three times a week, or at least once a day), volume of leakage (none or small amount, moderate amount, or large amount), and “How much does urine leakage affect your daily life?” (0 = not at all; 1–3 = mildly; 4–6 = moderately; 7–9 = severely; 10 = to a great deal). Based on the original scoring system, UI severity is categorized as slight (1–5), moderate (6–12), and severe to very severe (13–21). The Incontinence Impact Questionnaire-short form (IIQ-7), also validated in Arabic, was used to evaluate the effect of PPUI on quality of life (17, 20). This instrument evaluates seven activities: daily households chores (e.g., cooking, cleaning, and laundry), physical recreation (e.g., swimming, walking, and exercise), entertainment activities (e.g., movies and concerts), ability to travel by car or bus for more than 30 min, social activities outside the home, emotional health (e.g., nervousness and depression), and feelings of frustration. Participants were asked not to fill out the questionnaire more than once. The questionnaires were distributed electronically via a Google Forms link through social media applications, including X, Instagram, and WhatsApp.

### 2.2 Pilot study

A professional was consulted to evaluate whether the questionnaires were appropriate for the study’s objectives. A pilot study was conducted involving 20 participants to validate the questionnaires and ensure clarity. Participants in the pilot study were excluded from the final analysis. Written informed consent was obtained electronically from all participants prior to completing the survey.

### 2.3 Statistical analysis

Data were analyzed via SPSS version 25 statistical software. Descriptive statistics, including frequencies and percentages, were used to describe quantitative and categorical variables. Pearson’s chi-squared test assessed any associations between independent variables and outcome variables, such as seeking care, risk factors, and practices. A *p-*value of ≤ 0.05 and a 95% confidence interval (CI) were considered statistically significant. Univariate and multivariate regression analyses were performed to identify associations between independent variables and the likelihood of seeking care. Any variables in the univariate analysis with a *p*-value ≤ 0.2 were included in the multivariate model, while variables with higher *p*-values were excluded. Accordingly, employment status, chronic disease, and severity of UI were included in the multivariate analysis. Odds ratios were assessed with 95% confidence intervals.

## 3 Results

A total of 813 participants were enrolled in the study. The sociodemographic characteristics are summarized in Table 1. The largest age group was 35 to 44 years, comprising 37.5% (*n =* 305) of the participants. The majority of women were married (92.5%, *n =* 752), and 58.5% (*n =* 476) had a bachelor’s degree. Regarding employment status, 52.5% (*n =* 427) were housewives, while 28.7% (*n =* 233) were employed in the education sector.

**Table 1:** Sample characteristics of Saudi Arabian women who participated in a survey of postpartum urinary incontinence (*N =* 813).

Variables	Levels	*N*	%
Age (in years)	25–34 35–44 45–54 ≥55	237 305 223 48	29.2 37.5 27.4 5.9
Marital status	Married Divorced Widowed	752 31 30	92.5 3.8 3.7
Education level	High school Bachelor Post bachelor	272 476 65	33.5 58.5 8.0
Employment	None Health care Education Management Other	427 27 233 71 55	52.5 3.3 28.7 8.7 6.8
BMI (kg/m^2^)	Normal:18.5–24.9 Overweight: 25–29.9 Obese: ≥30	240 296 277	29.5 36.4 34.1
Number of children	1–2 3–4 More than 4	218 303 292	26.8 37.3 35.9
Types of delivery	Vaginal Cesarian Mixed	521 110 182	64.1 13.5 22.4
Exercise frequency (# of time/week)	Never 1–2 3 or More	417 280 116	51.3 34.4 14.3
Have chronic disease	No Yes	613 200	75.4 24.6
Type of chronic disease	No chronic disease Diabetes Hypertension Other	613 55 27 118	75.4 6.8 3.3 14.5
Experienced postpartum urinary incontinence	No Yes	572 241	70.4 29.6
Severity of urinary incontinence (based on classification of ICIQ)^a^	Slight Moderate Severe Very severe	57 122 49 13	23.7 50.7 20.7 5.4

Mixed delivery: more than one delivery; some are vaginal while others are cesarean. BMI: body mass index; other chronic diseases: asthma, thyroid gland disturbance, eczema, arteriosclerosis. ^a^ Sample size for the severity variable was *n =* 241.

The majority of participants were either overweight or obese (70.5%, *n =* 573), while 29.5% (*n =* 240) had a normal BMI. In terms of children, 37.3% (*n =* 303) of women had three to four children, and 35.9% had more than four children. Vaginal deliveries were reported by 64.1% (*n =* 521) of the respondents. Regarding physical activity, more than half of the respondents reported never exercising (51.3%), while 34.4% exercised one or two times per week. The majority of participants (75.4%, *n =* 613) did not report any chronic diseases, although 6.8% had diabetes. Among the 241 women (29.6%) who reported experiencing PPUI, 50.7% (*n =* 122) experienced it to a moderate degree, while 20.7% experienced severe PPUI, according to the International Consultation on Incontinence Questionnaire – Urinary Incontinence Short Form (ICIQ-UI SF) classification (18, 19).

**Figure 1:**
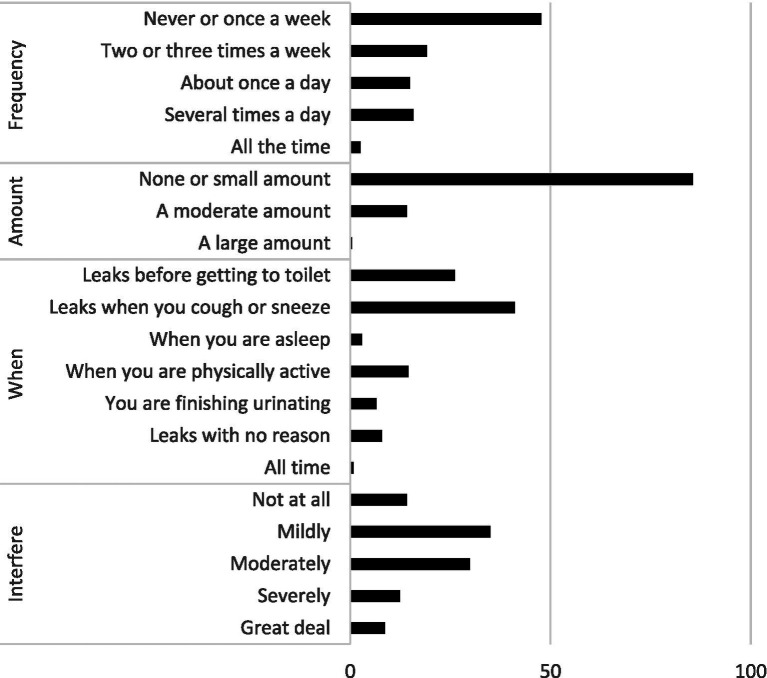
Assessment of frequency, amount, timing, and interference of postpartum urinary incontinence on daily life using the international consultation on incontinence questionnaire (*n =* 241).

Figure 1 showed that 15.8% (*n =* 38) of participants with PPUI experienced urine leakage several times a day, and 14.1% (*n =* 34) reported a moderate amount of leakage. Regarding the circumstances of leakage, 26.1% experienced leakage before reaching the toilet, while 41.1% experienced it during coughing or sneezing. Over 20% of respondents believed that PPUI severely or significantly interfered with their daily lives. As shown in Table 2, 56.5% (*n =* 459) of respondents incorrectly believed that PPUI is a normal condition after delivery, and approximately two-thirds (67.3%) believed it is a temporary condition that resolves over time. Most respondents correctly identified that the number of children (71.3%), presence of chronic diseases (62.5%), obesity (53.5%), and exercise (47.1%) were potential contributors to PPUI. However, 52.9% of women did not believe that exercise impacts urinary incontinence.

**Table 2:** Knowledge about postpartum urinary incontinence among Saudi women (*N =* 813).

Knowledge	Yes, *n* (%)	No, *n* (%)
Leaking urine after delivery is normal.	459 (56.5)	354 (45.5)*
Urinary incontinence is a temporary condition and disappears with time.	547 (67.3)	266 (32.7)*
The number of childbirths has an effect on urinary incontinence.	580 (71.3)*	233 (28.7)
There is a relationship between urinary incontinence and chronic diseases such as hypertension and diabetes mellitus.	508 (62.5)*	305 (37.5)
Obesity is related to urinary incontinence.	435 (53.5)*	378 (46.5)
Exercise has an impact on urinary incontinence.	383 (47.1)*	430 (52.9)
There is a relationship between urinary incontinence and depression, anxiety, and stress.	485 (59.7%)*	328 (40.3%)

*Indicates correct response.

**Figure 2:**
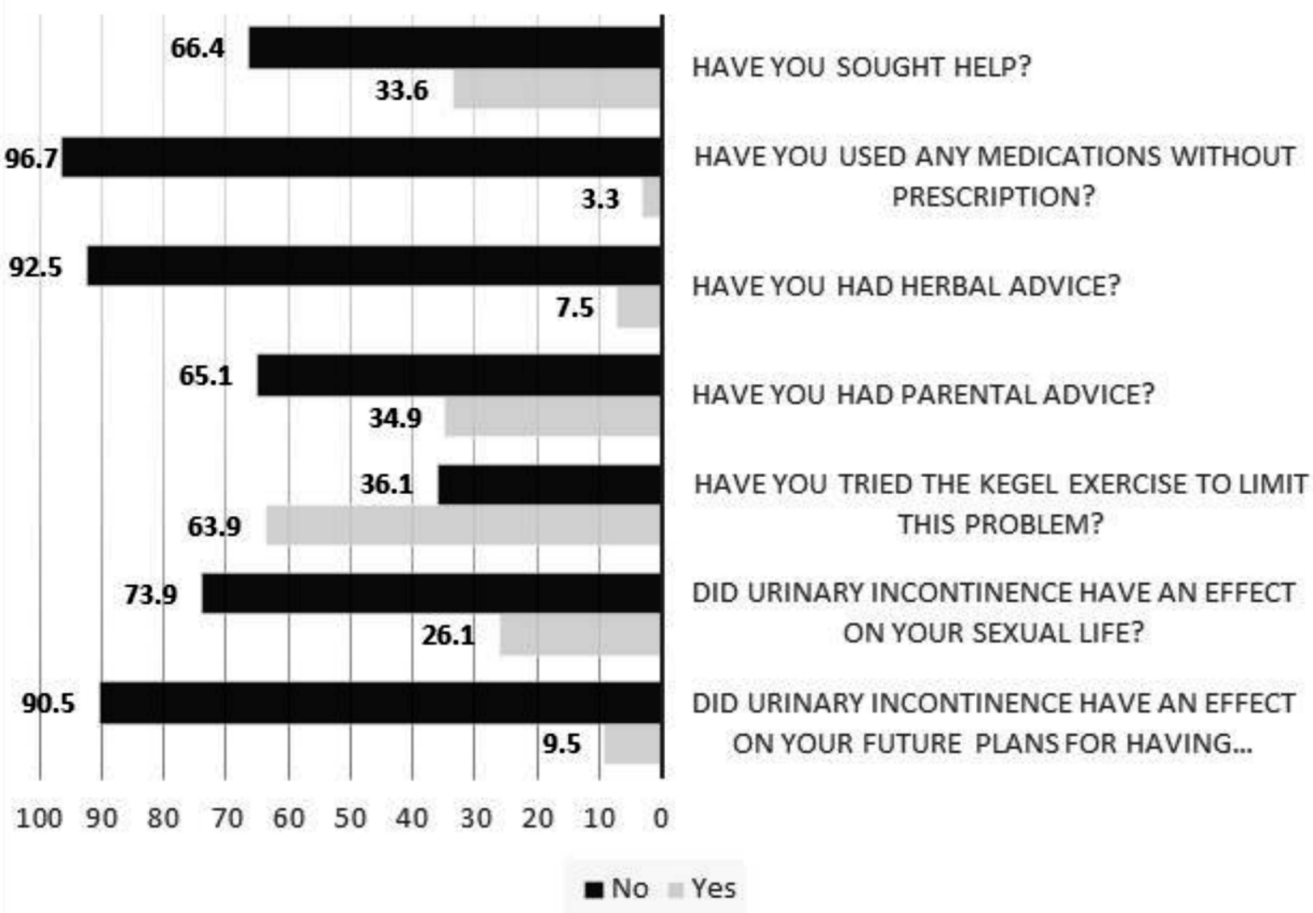
Frequency, experience, and impact of postpartum urinary incontinence on Saudi women with *n =* 241.

Figure 2 describes respondents’ practices related to PPUI. The majority (66.4%) did not seek professional medical care for their symptoms, while 34.9% sought parental advice. A substantial proportion (63.9%) reported attempting Kegel exercises to manage the condition; however, 52.7% of those who practiced them found the exercises to be ineffective. Despite experiencing PPUI, most participants reported that the condition did not impact their sexual life (73.9%) or their plans for having more children (90.5%).

As shown in Table 3, a statistically significant positive association was found between employment status and care-seeking behavior, with 42.9% of employed respondents seeking care compared to 38.4% of unemployed respondents (*p* = 0.003). Additionally, the severity of PPUI symptoms was significantly associated with care-seeking behavior (*p =* 0.003); among those who sought care, 56.1% reported experiencing severe or extremely severe symptoms. The presence of chronic diseases showed a trend toward association with seeking care (*p =* 0.08). Table 4 shows univariate logistic regression models for the independent variables. The severity of PPUI was significantly associated with seeking care (moderate: odds ratio = 4.4; severe: odds ratio = 1.8; *p* = 0.003). In multivariable regression analysis, variables including employment status, chronic diseases, and PPUI severity were included (Table 5). The analysis revealed that the severity of PPUI was significantly associated with seeking care (moderate: odds ratio = 4.5; severe: odds ratio = 1.7; *p* = 0.002). As shown in Table 6, PPUI interfered with daily life for more than 50% of participants. Additionally, 24% reported that PPUI greatly affected their ability to travel, while over 39% reported a significant impact on their participation in social activities outside the home.

**Table 3:** Comparison of characteristics between Saudi women who sought care for their urinary incontinence and those who did not (*n =* 241).

Variables	Levels	*N*	Sought care		Chi-squared *p*-value
			No (*n =* 160) *n* (%)	Yes (*n =* 81) *n* (%)	
Age (in years)	25–34 35–44 45–54 ≥55	58 85 81 17	38 (64.3%) 57 (67.1%) 55 (67.9%) 10 (56.3%)	20 (35.7%) 28 (32.9%) 26 (32.1%) 7 (43.8%)	0.781
Marital status	Married Divorced Widowed	219 11 11	148 (67.6%) 5 (45.5%) 7 (63.6%)	71 (32.4%) 6 (54.5%) 4 (36.4%)	0.311
Education level	High school Bachelor Post bachelor	78 148 15	46 (59.0%) 104 (70.3%) 10 (66.7%)	32 (41.0%) 44 (29.7%) 5 (33.3%)	0.232
Employed	No Yes	125 116	77 (61.6%) 83 (57.0%)	48 (38.4%) 33 (42.9%)	0.003*
BMI (kg/m^2^)	Normal Overweight Obese	50 81 110	37 (73.5%) 51 (63.0%) 72 (66.4%)	13 (26.5%) 30 (37.0%) 38 (33.6%)	0.682
Number of children	1–2 3–4 More than 4	48 86 107	34 (70.8%) 56 (65.1%) 70 (65.4%)	14 (29.2%) 30 (34.9%) 37 (34.6%)	0.766
Types of delivery	Vaginal Cesarian Mixed	158 23 60	102 (64.6%) 17 (73.9%) 41 (68.3%)	56 (35.4%) 6 (26.1%) 19 (31.7%)	0.630
Exercise frequency (# of times/week)	Never 1–2 3 or more	125 90 26	84 (67.2%) 60 (66.7%) 16 (51.6%)	41 (32.8%) 30 (33.3%) 10 (48.3%)	0.377
Have chronic disease	No Yes	173 68	120 (69.4%) 40 (58.8%)	53 (30.6%) 28 (41.2%)	0.080
Severity of urinary incontinence	Slight Moderate Severe to very severe	57 122 62	47 (85.1%) 81 (66.4%) 32 (43.9%)	10 (14.9%) 41 (33.6%) 30 (56.1%)	0.003*

*Significant *p* ≤ 0.05.

**Table 4:** Univariate logistic regression models in Saudi women who sought care for their urinary incontinence (*n =* 241).

Variable	Level	OR	95% confidence limits	*p*-value
Age (in years)	18–34 35–44 45–54 ≥55	Ref 1.330 1.425 1.481	0.440–4.025 0.490–4,140 0.507–4.328	0.907
Marital status	Married Divorced Widowed	Ref 1.191 0.476	0.338–4.202 0.086–2.628	0.332
Education level	High school Bachelor Post bachelor	Ref 0.719 1.182	0.224–2.303 0.382–3.658	0.235
Employed	No Yes	Ref 0.638	0.371–1.095	0.103
BMI (kg/m^2^)	Normal Overweight Obese	Ref 1.502 0.897	0.714–3.161 0.493–1.632	0.417
Number of children	1–2 3–4 More than 4	Ref 1.284 0.987	0.613–2.688 0.544–1.791	0.767
Types of delivery	Vaginal Cesarian Mixed	Ref 0.844 1.313	0.448–1.591 0.447–3.859	0.633
Exercise frequency (# of time/week)	Never 1–2 ≥3	Ref 4.098 4,111	0.721–23.298 0.719–23.504	0.274
Have chronic disease	No Yes	Ref 1.585	0.886–2.834	0.120
Severity of urinary incontinence	Slight Moderate Severe to very severe	Ref 4.406 1.852	1.893–10.257 0.992–3.456	0.003*
Knowledge about urinary incontinence	Q1 Q2 Q3	Ref 0.949 1.316	0.505–1.782 0.640–2.705	0.615

*Significant.

**Table 5:** Multivariate logistic regression analysis of factors associated with care-seeking among Saudi women with urinary incontinence (*n =* 241).

Variable	Level	OR	95% confidence limits	*p*-value
Employed	No Yes	Ref 0.505	0.339–1.043	0.070
Have chronic disease	No Yes	Ref 1.473	0.803–2.834	0.210
Severity of urinary incontinence	Slight Moderate Severe to very severe	Ref 4.595 1.784	1.947–10.845 0.945–3.368	0.002*

*Significant. Variables with a *p* ≤ 0.2 in the univariate analysis were included in the multivariate model, while variables with higher *p*-values were excluded.

**Table 6:** Impact of postpartum UI on activities, relationships, and feelings on saudi women [incontinence impact questionnaire, short form (IIQ-7)], *n =* 241.

Variable		*N*	(%)
Ability to do household chores (cooking, housecleaning, and laundry)	Not at all Slightly Moderately Greatly	97 38 61 45	(40.2) (15.8) (25.3) (18.7)
Physical recreation such as walking, swimming, or other exercise?	Not at all Slightly Moderately Greatly	91 63 61 26	(37.8) (26.1) (25.3) (10.8)
Entertaining activities (movies, concerts, etc.)	Not at all Slightly Moderately Greatly	115 66 39 21	(47.7) (27.7) (16.2) (8.7)
Ability to travel by car or bus more than 30 min from home	Not at all Slightly Moderately Greatly	84 55 44 58	(34.9) (22.8) (18.3) (24)
Participation in social activities outside your home	Not at all Slightly Moderately Greatly	91 54 54 42	(37.8) (22.4) (22.4) (17.4)
Emotional health (nervousness, depression, etc.)	Not at all Slightly Moderately Greatly	82 63 73 23	(34.0) (26.1) (30.3) (9.5)
Feeling frustrated	Not at all Slightly Moderately Greatly	105 86 36 14	(43.6) (35.7) (14.9) (5.8)

## 4 Discussion

Our study found a prevalence of PPUI of 29.6% among postpartum Saudi women, which is lower than rates reported in Türkiye (35.9%) and the Netherlands (57.1%) (11, 21). However, a systematic review reported a slightly higher prevalence (33%) (12). In our sample, participants also reported higher symptom severity, with 50.7% experiencing moderate and 20.7% experiencing severe PPUI symptoms. This contrasts with findings from Hsieh et al., in which only 33% of participants considered their PPUI to be bothersome (21). However, that study was conducted in Taiwan, a population with a different cultural and ethnic background. The higher reported severity among our participants may help explain why over 20% of respondents reported that PPUI severely or greatly interfered with their daily lives.

An important finding in our study was the low level of knowledge about PPUI among participants. Notably, 56.5% of respondents believed that PPUI is a normal outcome of childbirth. The relatively high prevalence of PPUI may contribute to the widespread perception that it is an expected consequence of delivery. One barrier to seeking help is the misconception that PPUI is a normal part of aging (17). Interestingly, the majority of participants (71.3%) associated increased PPUI with the number of children, an observation likely drawn from personal experience. Additionally, nearly half of the respondents believed that chronic diseases, obesity, and lack of exercise may influence the development of PPUI. In our study, 66.4% of women with PPUI did not seek medical care. Multiple cultural and regional factors can act as barriers to seeking medical attention. For instance, a study conducted in Egypt reported that 80% of women did not pursue medical advice, primarily due to embarrassment, lack of awareness about the symptoms, and limited knowledge of available treatment options (17). Similarly, in the Netherlands, 75% of women did not seek help, assuming that their symptoms would resolve on their own and were not severe enough to require medical attention (22). Feelings of embarrassment and shyness have also been reported as reasons for not seeking help among postmenopausal women in other studies (21, 23). A study from France found that 60.3% of women never reported their symptoms to a physician, and that age, BMI, and parity were associated with UI (24). Other factors, such as social support, individual needs, and access to healthcare services, have also been identified to be associated with care-seeking behavior among postpartum women with UI (25). These findings highlight considerable variation in care-seeking behavior across different populations. Although more than 66% of our participants held a bachelor’s or postgraduate degree, a significant proportion (over 75%) experienced moderate to severe PPUI, yet overall knowledge and awareness of the condition remained low. This underscores the crucial role of healthcare professionals in educating women about PPUI and encouraging its appropriate management and treatment.

It is understandable, as PPUI may be more disruptive in an occupational setting compared to the relative privacy and flexibility of the home environment. Full-time employment can exacerbate the challenges of managing PPUI, leading working women to seek medical assistance more frequently (26). While individuals often tolerate mild symptoms, especially when busy with work, the severity of PPUI remains a primary driver for seeking medical care. Additionally, concerns about potential complications from chronic diseases may prompt individuals to seek care, as suggested by the marginally significant association observed between chronic diseases and care-seeking behavior. However, our findings did not show any correlation between employment and PPUI.

With regard to Kegel exercises, the majority of respondents appeared to have received information from healthcare providers, as these exercises are a routine component of postpartum care in Saudi Arabia. Consequently, 63.9% of participants reported practicing Kegel exercises to alleviate PPUI symptoms. However, 52.7% of those who attempted them perceived the exercises as ineffective. In contrast, other studies have reported that 45 to 80% of women do not receive health advice related to PPUI (27, 28). Notably, sexual life (26%) and future childbearing intentions (9.5%) were not affected by PPUI among our participants, likely reflecting cultural preferences in Arab societies that favor larger families.

We found that nearly 70% of participants were aged 35 years or older, consistent with previous studies reporting a positive association between age and the occurrence of PPUI. This relationship may be attributed to the age-related weakening of the pelvic floor muscles (29). Additionally, our findings revealed that approximately one-third of the participants (34.1%) were classified as obese, and BMI was positively associated with PPUI. Increased body weight likely exerts greater pressure on the bladder, contributing to urinary symptoms. Moreover, more than 64% of our participants had undergone vaginal delivery, which aligns with most studies demonstrating a positive correlation between the mode of delivery and the risk of developing PPUI (14, 30). On the other hand, our findings did not show a significant association between chronic diseases and PPUI, in contrast to results reported in other studies (14, 31).

PPUI significantly impacts patients’ quality of life (32). Our findings indicated that PPUI interfered with daily activities for more than 50% of respondents. Despite this, over 66% did not seek medical care, highlighting the need for increased awareness and intervention. Approximately 24% of participants reported that PPUI greatly affected their ability to travel. Cultural factors may contribute to this, as many women find it difficult or uncomfortable using public restrooms, such as those found in transportation hubs or along highways. As a result, some women may alter their travel plans to avoid the inconvenience associated with incontinence. Furthermore, we recently identified a significant association between PPUI and psychological conditions, including depression, anxiety, and stress, highlighting its detrimental effects on mental health and overall wellbeing (33). The majority of participants in our study experienced urine leakage after coughing or sneezing, which is characteristic of stress urinary incontinence, the subtype most commonly linked to pregnancy and childbirth. However, it is possible that symptoms are attributable to both stress and urgency incontinence, a condition referred to as mixed incontinence (34, 35).

### 4.1 Limitations

This study has several limitations. Cross-sectional studies are unable to infer causality. As a questionnaire-based investigation, the study is susceptible to recall bias, as participants may not accurately remember past events or may be reluctant to report their symptoms. Additionally, the reliability of self-reported data is inherently limited, and such data cannot establish a causal relationship. Online sampling may also introduce bias, favoring women with greater digital access and potentially higher levels of education. Furthermore, older and rural women, as well as those with limited access to digital platforms, are likely to be underrepresented. Age and the time interval since the relevant events may also influence participants’ responses. Considering these factors, the findings of this study cannot be generalized to represent the disorder at the national level. Therefore, a large-scale study conducted across different regions and settings would be of great value. Finally, different types of PPUI cannot be considered homogeneous due to underlying pathophysiological and anatomical differences among patients, which may lead to variability in symptom perception and response.

## 5 Conclusion

This study identified a high prevalence of Saudi women who did not seek care for PPUI, with a positive correlation between seeking care and symptom severity. Additionally, a lack of accurate knowledge about PPUI and its risk factors was observed even among highly educated women, indicating a gap in health education and awareness. Over half of the women reported that PPUI interfered with their daily lives. These findings underscore the need for comprehensive educational programs targeting both patients and healthcare providers. Effective counseling by healthcare professionals during postpartum discharge, along with inclusion of the Kegel exercises in treatment plans and follow-up visits, is essential to improve awareness and encourage timely intervention based on the underlying causes of PPUI. Such measures can help reduce the burden and complications of PPUI among Saudi women.

## Data Availability

The original contributions presented in the study are included in the article/supplementary material, further inquiries can be directed to the corresponding author.

## References

[1] Minassian VA Drutz HP Al-Badr A. Urinary incontinence as a worldwide problem. Int J Gynaecol Obstet. (2003) 82:327–38. doi: 10.1016/s0020-7292(03)00220-0, PMID: 14499979

[2] Al-Badr A Brasha H Al-Raddadi R Noorwali F Ross S. Prevalence of urinary incontinence among Saudi women. Int J Gynaecol Obstet. (2012) 117:160–3. doi: 10.1016/j.ijgo.2011.12.014, PMID: 22356760

[3] Altaweel W Alharbi M. Urinary incontinence: prevalence, risk factors, and impact on health related quality of life in Saudi women. Neurourol Urodyn. (2012) 31:642–5. doi: 10.1002/nau.22201, PMID: 22415626

[4] Abduldaiem A AlIssa H Saleim M Kofi M. Prevalence and risk factors of urinary incontinence among adult Saudi women in Riyadh, Saudi Arabia. Int J Med and Health Res. (2020) 6:56–64.

[5] Al Kiyumi MH Al Belushi ZI Jaju S Al Mahrezi AM. Urinary incontinence among Omani women: prevalence, risk factors and impact on quality of life. Sultan Qaboos Univ Med J. (2020) 20:e45–53. doi: 10.18295/squmj.2020.20.01.007 32190369 PMC7065693

[6] The Office on Women's Health. (2019). Urinary incontinence. Available online at: https://www.womenshealth.gov/a-z-topics/urinary-incontinence (accessed January 31, 2019).

[7] Doumouchtsis SK De Tayrac R Lee J Daly O Melendez-Munoz J Lindo FM . An international continence society (ICS)/international Urogynecological association (IUGA) joint report on the terminology for assessment and management of obstetric pelvic floor disorders. Continence. (2022) 22:100502. doi: 10.1016/j.cont.2022.100502

[8] Harland N Walz S Eberli D Schmid FA Aicher WK Stenzl A . Stress urinary incontinence: an unsolved clinical challenge. Biomedicine. (2023) 11:2486. doi: 10.3390/biomedicines11092486, PMID: 37760927 PMC10525672

[9] Diez-Itza I. Urinary incontinence during pregnancy and in the postpartum period. Nat Rev Urol. (2025) 22:1091. doi: 10.1038/s41585-025-01091-x, PMID: 41053483

[10] Meekins AR Siddiqui NY. Diagnosis and management of postpartum pelvic floor disorders. Obstet Gynecol Clin N Am. (2020) 47:477–86. doi: 10.1016/j.ogc.2020.05.002, PMID: 32762932

[11] Dinç A Oymak S Çelik M. Examining prevalence of urinary incontinence and risk factors in women in third postpartum month. Int J Urol Nurs. (2018) 13:13–22. doi: 10.1111/ijun.12176

[12] Thom DH Rortveit G. Prevalence of postpartum urinary incontinence: a systematic review. Acta Obstet Gynecol Scand. (2010) 89:1511–22. doi: 10.3109/00016349.2010.526188, PMID: 21050146

[13] Danforth KN Townsend M Lifford K Curhan GC Resnick NM Grodstein F. Risk factors for urinary incontinence among middle-aged women. Am J Obstet Gynecol. (2006) 194:339–45. doi: 10.1016/j.ajog.2005.07.051, PMID: 16458626 PMC1363686

[14] Chang SR Lin WA Chang TC Lin HH Lee CN Lin MI. Risk factors for stress and urge urinary incontinence during pregnancy and the first year postpartum: a prospective longitudinal study. Int Urogynecol J. (2021) 32:2455–64. doi: 10.1007/s00192-021-04788-w, PMID: 33835213

[15] Almutairi S Alobaid O Al-Zahrani MA Alkhamees M Aljuhayman A Ghazwani Y. Urinary incontinence among Saudi women: prevalence, risk factors, and impact on quality of life. Eur Rev Med Pharmacol Sci. (2021) 25:6311–8. doi: 10.26355/eurrev_202110_27001, PMID: 34730211

[16] Wagg AR Kendall S Bunn F. Women's experiences, beliefs and knowledge of urinary symptoms in the postpartum period and the perceptions of health professionals: a grounded theory study. Prim Health Care Res Dev. (2017) 18:448–62. doi: 10.1017/S1463423617000366, PMID: 28825530

[17] El-Azab AS Shaaban OM. Measuring the barriers against seeking consultation for urinary incontinence among middle eastern women. BMC Womens Health. (2010) 10:3. doi: 10.1186/1472-6874-10-3, PMID: 20105307 PMC2835642

[18] Avery K Donovan J Peters TJ Shaw C Gotoh M Abrams P. ICIQ: a brief and robust measure for evaluating the symptoms and impact of urinary incontinence. Neurourol Urodyn. (2004) 23:322–30. doi: 10.1002/nau.20041, PMID: 15227649

[19] Al-Shaikh G Al-Badr A Al Maarik A Cotterill N Al-Mandeel HM. Reliability of Arabic ICIQ-UI short form in Saudi Arabia. Urol Ann. (2013) 5:34–8. doi: 10.4103/0974-7796.106964, PMID: 23662008 PMC3643321

[20] Shumaker SA Wyman JF Uebersax JS McClish D Fantl JA. Health-related quality of life measures for women with urinary incontinence: the incontinence impact questionnaire and the urogenital distress inventory. Qual Life Res. (1994) 3:291–306. doi: 10.1007/BF00451721, PMID: 7841963

[21] Hsieh CH Su TH Chang ST Lin SH Lee MC Lee MY. Prevalence of and attitude toward urinary incontinence in postmenopausal women. Int J Gynaecol Obstet. (2008) 100:171–4. doi: 10.1016/j.ijgo.2007.08.013, PMID: 17977542

[22] Alkishi NA Akishi AM Althabit FM Alali HA Alyousof AA. Prevelence of urinary incontinence in Al Ahsa region Saudi Arabia. Int. J. Med. Dev. Countries. (2022) 6:1–5. doi: 10.24911/IJMDC.51-1614107061

[23] Moossdorff-Steinhauser HFA Berghmans BCM Spaanderman MEA Bols EMJ. Urinary incontinence 6 weeks to 1 year post-partum: prevalence, experience of bother, beliefs, and help-seeking behavior. Int Urogynecol J. (2021) 32:1817–24. doi: 10.1007/s00192-020-04644-3, PMID: 33484286 PMC8295159

[24] Lasserre A Pelat C Guéroult V Hanslik T Chartier-Kastler E Blanchon T . Urinary incontinence in French women: prevalence, risk factors, and impact on quality of life. Eur Urol. (2009) 56:177–83. doi: 10.1016/j.eururo.2009.04.006, PMID: 19376639

[25] Liang S Chen Z Tang W Andarini E Kou L Li Y . Prevalence and predictors of help-seeking behavior among post-partum women with urinary incontinence in China and Indonesia: a cross-sectional survey based on Andersen help-seeking model. Midwifery. (2024) 128:103885. doi: 10.1016/j.midw.2023.103885, PMID: 37984080

[26] Chang SR Lin WA Lin HH Lee CN Chang TC Lin MI. Cumulative incidence of urinary incontinence and associated factors during pregnancy and after childbirth: a cohort study. Int Urogynecol J. (2022) 33:1451–61. doi: 10.1007/s00192-021-05011-6, PMID: 34783862

[27] Tantisiriwat N Manchana T. Knowledge and acceptance of Thai women toward the pelvic floor muscle training. J Med Assoc Thail. (2014) 97:7–11. PMID: 24701723

[28] Hermansen IL O'Connell B Gaskin CJ. Are postpartum women in Denmark being given helpful information about urinary incontinence and pelvic floor exercises? J Midwifery Womens Health. (2010) 55:171–4. doi: 10.1016/j.jmwh.2009.09.004, PMID: 20189136

[29] Barbosa L Boaviagem A Moretti E Lemos A. Multiparity, age and overweight/obesity as risk factors for urinary incontinence in pregnancy: a systematic review and meta-analysis. Int Urogynecol J. (2018) 29:1413–27. doi: 10.1007/s00192-018-3656-9, PMID: 29754281

[30] Alshehri SZ Abumilha AK Amer KA Aldosari AA Shawkhan RA Alasmari KA . Patterns of urinary incontinence among women in Asir region, Saudi Arabia. Cureus. (2022) 14:e21628. doi: 10.7759/cureus.21628 35233309 PMC8881247

[31] Rocha J Brandão P Melo A Torres S Mota L Costa F. Assessment of urinary incontinence in pregnancy and postpartum: observational study. Acta Medica Port. (2017) 30:568–72. doi: 10.20344/amp.7371, PMID: 28926331

[32] Rodríguez-Almagro J Hernández Martínez A Martínez-Vázquez S Peinado Molina RA Bermejo-Cantarero A Martínez-Galiano JM. A qualitative exploration of the perceptions of women living with pelvic floor disorders and factors related to quality of life. J Clin Med. (2024) 13:1896. doi: 10.3390/jcm13071896, PMID: 38610661 PMC11012559

[33] Al-Khlaiwi T Habib SS Saquib N Muaddi S Alotaibi S Hadba AB. The associations of postpartum urinary incontinence with depression, anxiety, and stress among women in Saudi Arabia: a cross-sectional study. BMC Psychol. (2025) 13:412. doi: 10.1186/s40359-025-02734-9, PMID: 40259357 PMC12013183

[34] Jansson MH Franzén K Tegerstedt G Hiyoshi A Nilsson K. Stress and urgency urinary incontinence one year after a first birth-prevalence and risk factors. A prospective cohort study. Acta Obstet Gynecol Scand. (2021) 100:2193–201. doi: 10.1111/aogs.14275, PMID: 34699060

[35] Haylen BT de Ridder D Freeman RM Swift SE Berghmans B Lee J . International Urogynecological association; international continence society. An international Urogynecological association (IUGA)/international continence society (ICS) joint report on the terminology for female pelvic floor dysfunction. Neurourol Urodyn. (2010) 29:4–20. doi: 10.1002/nau.20798, PMID: 19941278

